# Responses of Rice Yield, N Uptake, NH_3_ and N_2_O Losses from Reclaimed Saline Soils to Varied N Inputs

**DOI:** 10.3390/plants12132446

**Published:** 2023-06-25

**Authors:** Si Wu, Zhenhua Zhang, Haijun Sun, Haibo Hu

**Affiliations:** 1 Co-Innovation Center for Sustainable Forestry in Southern China, College of Forestry, Nanjing Forestry University, Nanjing 210037, China; nlwus@njfu.edu.cn (S.W.); hhb@njfu.com.cn (H.H.); 2 Jiangsu Key Laboratory for Bioresource of Saline Soils, School of Wetlands, Yancheng Teachers University, Yancheng 224007, China; zhenhua.zhang@uwa.edu.au; 3 School of Agriculture and Environment, The University of Western Australia, Crawley, WA 6009, Australia

**Keywords:** saline soil, N fertilizer, rice production, NH_3_ volatilization, N_2_O emissions

## Abstract

It is of agronomic importance to apply nitrogen (N), but it has high environmental risks in reclaimed saline soils. Therefore, we should apply N fertilizer at an appropriate rate to increase crop yield but decrease N losses. In this soil column experiment, rice yield, N uptake, and ammonia (NH_3_) and nitrous oxide (N_2_O) losses were measured in four treatments with no N application (control) and with N applications of 160, 200, and 240 kg/ha (N160, N200, and N240, respectively). The results show that grain yield, spike number, and thousand-kernel weight increased with increases in N application rate, but there was no significant difference in grain yield between N200 and N240. However, the kernels per spike increased first and then decreased with the increase in N application, of which N200 was recorded to have the highest kernels per spike value, which was 16.8 and 9.8% higher than those of N160 and N240, respectively. Total NH_3_ volatilization of the rice season increased with increasing N input, especially during the first and second supplementary fertilization stages. The NH_4_^+^-N concentration of overlying water was relatively lower under the N200 treatment in these two stages, and the yield-scaled NH_3_ volatilization and the emission factor were the lowest in N200, which were 26.2–27.8% and 4.0–21.0% lower than those of N160 and N240, respectively. Among the three N-applied treatments, N_2_O losses and the emission factor as well as the yield-scaled N_2_O emissions were the lowest under the N200 treatment, which had 34.7% and 78.9% lower N_2_O emissions and 57.8% and 83.5% lower emission factors than those of the N160 and N240 treatments, respectively. Moreover, the gene copies of AOA and AOB *amoA*, *nirS*, and *nirK* in cultivated layer soils all reached the minimum under the N200 treatment. According to the comprehensive effects of N fertilizer on rice grain yield and NH_3_ and N_2_O losses, we recommend applying 200 kg/ha to reclaimed saline soil to ensure crop yield and reduce N losses.

## 1. Introduction

In recent years, China’s cultivated land has been declining, with 0.01% of cultivated land being destroyed every year. It is estimated that China’s per capita cultivated land area will decrease to less than 0.08 ha between 2010 and 2030, which will become a significant challenge for sustainable development and food security [1,2]. Although China attaches great importance to cultivated land protection, the quantity and quality of cultivated land are still declining, and the conflict between population and cultivated land resources is becoming increasingly significant [3]. Therefore, there is an urgent need to develop and utilize other land resources to improve the current situation of cultivated land reduction. Saline soil is an important reserve land resource in grain production, and more than 800 million hectares of cultivated land around the world are affected by salinization. In China, saline land accounts for 25% of the land, and these land resources have not been fully developed and expanded [4,5]. Therefore, rational utilization of saline soil resources is of great significance. It is well known that the soil nutrients of reclaimed saline soil are generally poor, and nitrogen (N) is the nutrient that limits the yield and is the most difficult to manage in the crop system of saline soil. Hence, it is necessary to increase the N nutrient content in the processes of developing and utilizing saline soil [6]. However, excessive N application is common in farmland management and food production, and a large amount of N loss causes a series of severe environmental problems, such as water pollution, intensified ammonia (NH_3_) volatilization, and increased nitrous oxide (N_2_O) emissions [7,8,9]. Thus, it is especially necessary to consider the rational application of N fertilizer in the processes of cultivation and crop planting in reclaimed saline soil [10].

Saline–alkaline soil is not suitable for farming because of its low fertility and high salt content, which results in the national food security strategy of “ask for grains from saline-alkaline land” requiring more effort [11]. Rice is one of the stable crops that can survive in saline soil, and its planation could help to improve saline soil fertility [12]. It is well known that gaseous N losses, such as N_2_O emissions and NH_3_ volatilization, have been the main forms of N losses in rice paddy fields [13]. In recent years, the area of rice planting in saline soil has gradually increased, and the study on yield and N losses in the process of rice planting in saline soil has also increased. Bao et al. (2019) conducted experiments on four kinds of salt-tolerant rice under different N application rates. The results showed that reasonable application of N in saline soils was beneficial to increasing the spike number and kernels per spike, and the yield of the four kinds of rice reached the maximum when the N application rate was 150 kg/ha [14]. With the increase in the N application rate, the chlorophyll content of rice also increased, and the maintenance time of the green leaf area was longer, which was conducive to rice photosynthesis and dry matter accumulation, thus improving grain yield [15]. However, Qiu et al. (2022) found that the application of N is the main factor that caused a significant increase in NH_3_ volatilization [16]. Urea hydrolyzes quickly in paddy fields, and NH_4_^+^-N concentration of the overlying water is highly consistent with the NH_3_ volatilization rate [17]. In a rice field experiment, Kim et al. (2021) showed that N_2_O emissions increased significantly with an increase in the N application rate [18]. In addition, in the saline soil area of Songnen plain, China, N application significantly promoted N_2_O emissions, which increased with the increase in the N application rate and reached the maximum when the N application rate was 250 kg/ha [19]. In alkaline soil, ammonia-oxidizing bacteria (AOB) play a dominant role in nitrification, but AOB do not contain N_2_O reductase, which may lead to an increase in N_2_O emissions under a high N input condition [20,21]. Liu et al. (2010) found that a lack of N fertilizer may reduce soil nitrate N (NO_3_^−^-N) and nitrite N contents, resulting in a lack of substrates for *nirK* and *nirS* denitrification microorganisms, and that a reduction in *nirK* and *nirS* gene abundance may also decrease N_2_O emissions [22].

In summary, there have been many studies on the effects of N application rate on the rice-production system in saline soil, but few studies have comprehensively considered the responses of rice yield, N uptake, and environmental losses based on different N application rates in saline soils. Therefore, a soil column experiment was conducted to comprehensively evaluate the effects of three N inputs on rice yield, NH_3_ volatilization, and N_2_O emissions, which could help to explore an optimal N application rate for rice production in saline soil. Under the optimal N input condition, we can save production costs while ensuring crop yield and reducing environmental N losses.

## 2. Results

### 2.1. Rice Growth, Yield, and the Related Agronomic Traits

**Figure 1:**
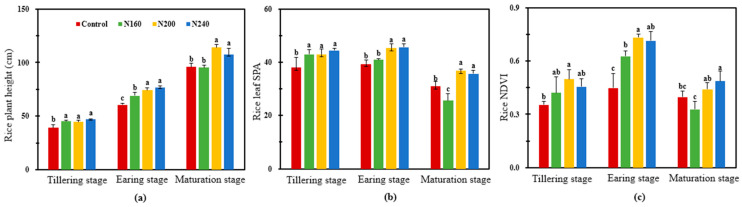
Response of the plant height (**a**), SPAD (**b**), and NDVI (**c**) of rice grown in saline soil to different N application rates. Bars refer to the SD of the mean value (*n* = 3). Different letters above the same column indicate statistically significant differences between treatments, according to Duncan’s post hoc test at the level of *p* < 0.05.

Compared with the control, N addition significantly (*p* < 0.05) increased the plant height, Soil Plant Development Analysis (SPAD), and Normalized Difference Vegetative Index (NDVI) at the tillering stage (Figure 1). At the earing stage, the plant height, leaf SPAD, and NDVI of rice increased with the increase in the N application rate, but there was no significant difference between the N200 and N240 treatments. At maturation stage, rice leaf SPAD in the N160 treatment was significantly (*p* < 0.05) lower than that in the control treatment. Nevertheless, rice plant height, leaf SPAD, and NDVI in the N200 and N240 treatments were higher than those in the control treatment, but there was no significant difference between the N160 and N240 treatments (Figure 1).

More N application resulted in significantly (*p* < 0.05) higher rice straw biomass, of which N200 significantly (*p* < 0.05) enhanced the rice grain yield by 61.3% over N160. However, the grain yield did not further increase when the N application rate continued to increase to 240 kg/ha, which might have been a result of its significantly (*p* < 0.05) lower harvest index than that of N160 and N200 (Table 1).

With the increase in the N application rate from 0 to 240 kg/ha, the spike number continuously significantly (*p* < 0.05) increased, but the kernels per spike increased first and then decreased, and reached the maximum under the N200 treatment, which was 36.3% and 9.8% higher than that in the control and N240 treatments, respectively. The thousand-kernel weight was slightly higher under the N200 and N240 treatments (Table 1).

### 2.2. N Content and Uptake of Rice Straw and Grain

The N content of rice straw increased with the increase in the N application rates, and the N content in the N200 and N240 treatments significantly (*p* < 0.05) increased by 53.5% and 62.6%, respectively, compared with the control (Table 2). However, the N content of grain increased first and then decreased with the increase in N inputs and reached the maximum in the N200 treatment, which was 11.0% and 6.0% (*p* < 0.05) higher than that in the N160 and N240 treatments, respectively. Generally, rice straw, grain, and their total N uptake increased with the increase in the N application rates. For the three N-added treatments, the N uptakes by rice straw and grain in N200 and N240 were on average 2.8- and 1.8-fold, respectively, that in N160. Consequently, total N uptake by rice plants in N200 and N240 was 1.9- and 2.3-fold that in N160 (Table 2).

### 2.3. NH_3_ Volatilization

With the increase in the N application rates, NH_3_ volatilization from rice paddies at the observations during the basal (BF) and first supplementary fertilizations (SF1) of N increased continuously, a pattern that was not observed after the second supplementary fertilization (SF2) (Table 3). Overall, the NH_3_ volatilization at the BF and SF2 observations was less than that at the SF1 observation. It was noted that a larger NH_3_ volatilization in the N240 treatment in the BF was recorded, which was 1.9 and 1.2 times higher than that of the N160 and N200 treatments, respectively. More N input resulted in higher total NH_3_ volatilization of the rice-growth cycle. In particular, the total NH_3_ volatilization was 17.1–68.6% (*p* < 0.05) higher in the N240 than in the N160 and N200 treatments, respectively. Under the three N-amended treatments, N200 had the lowest emission factor and yield-scaled NH_3_ volatilization, which were 27.8% and 26.1% (*p* < 0.05) lower than those in N160 and N240, respectively (Table 3).

### 2.4. Dynamics of pH, NH_4_^+^-N, and NO_3_^−^-N in Overlying Water

#### 2.4.1. pH

**Figure 2:**
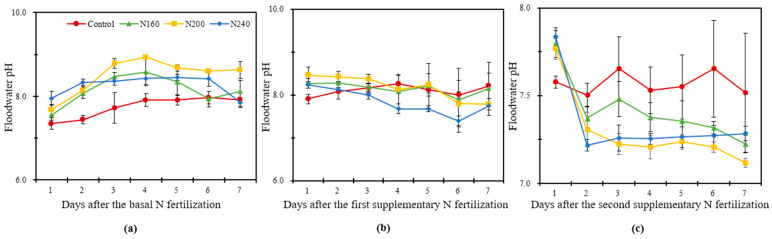
The variation in pH of the overlying water within one week after the basal N fertilization (**a**), first supplementary fertilization (**b**) and second supplementary fertilization (**c**) in saline soil with different N application rates. Bars refer to the SD of the mean value (*n* = 3).

The pH of the overlying water showed an overall trend, first increasing and then decreasing. After the third day of BF, the pH of the overlying water in N200 was always higher than that of the other treatments, with a maximum value of 8.9 (Figure 2a). The pH of the overlying water in N200 had the highest value throughout the BF, with an average increase of 0.34 and 0.24 units compared to N160 and N240, respectively. The pH of the overlying water did not change significantly during the SF1 observation, but the highest pH value was also recorded in N200, whereas the pH was always lower in the N240 treatment than in other treatments. The pH in the N application treatments dropped sharply on the second day after SF2 and then gradually fluctuated downwards. It was at its highest in the N160 treatment. The pH varied from 7.12 to 7.77 in the N200 treatment during SF2, which was higher than the range of variation in the other treatments. The overlying water pH was lower in the N200 treatments than in the other treatments after day 3 during SF2 (Figure 2c).

#### 2.4.2. Ammonium N in Overlying Water

**Figure 3:**
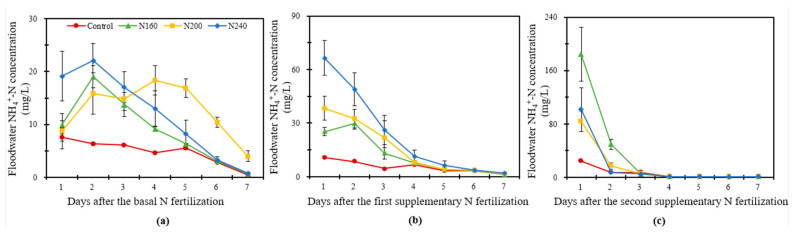
The variation in the NH_4_^+^-N concentration of the overlying water within one week after the basal N fertilization (**a**), first supplementary fertilization (**b**) and second supplementary fertilization (**c**) in saline soil with three N application rates. Bars refer to the SD of the mean value (*n* = 3).

As can be seen from Figure 3, the ammonium N (NH_4_^+^-N) concentration was significantly (*p* < 0.05) higher in the N-applied treatments than in the control treatment in the overlying water and showed an overall increasing trend followed by a decreasing trend thereafter. During BF, the NH_4_^+^-N concentration increased immediately and then decreased in the overlying water, with the NH_4_^+^-N concentration in the N160 and N240 treatments reaching the peak on the second day of fertilizer application; however, the peak was found on the fourth day in the N200 treatment. Compared with other treatments, the NH_4_^+^-N concentration in the overlying water was the highest in the N240 treatment during the first three days after N fertilizer application, whereas the NH_4_^+^-N concentration was always higher in the N200 treatment than in the other treatments after the fourth day of N fertilizer application. The average NH_4_^+^-N concentration was 44% and 7% higher in the N200 treatment than in the N160 and N240 treatments, respectively, during BF (Figure 3a). The NH_4_^+^-N concentration in the overlying water showed a consistently decreasing trend during SF1 and SF2. In SF1, the NH_4_^+^-N concentration in the overlying water decreased obviously from the first to the fourth days after fertilization and then decreased slowly (Figure 3b). In SF2, the NH_4_^+^-N concentration in the overlying water decreased sharply on the second day and then stabilized, gradually approaching almost 0 mg/L (Figure 3c). The highest NH_4_^+^-N concentrations in the SF1 and SF2 observations were both recorded in the N240 and N160 treatments, whereas the lowest NH_4_^+^-N concentration in SF2 was in the N200 treatment.

#### 2.4.3. NO_3_^−^-N in Overlying Water

**Figure 4:**
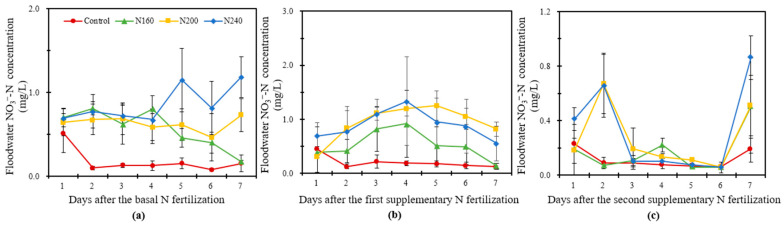
The variation in the NO_3_^−^-N concentration in the overlying water within one week after the basal N fertilization (**a**), first supplementary fertilization (**b**) and second supplementary fertilization (**c**) in saline soil with different N application rates. Bars refer to the SD of the mean value (*n* = 3).

Figure 4 shows that the NO_3_^−^-N concentration in the overlying water was generally low and that the overall range in variation was relatively small. In BF, the NO_3_^−^-N concentration in the overlying water increased in the N200 and N240 treatments but decreased in the N160 treatment with time. The N240 treatment had the highest value of average NO_3_^−^-N concentration (Figure 4a). The NO_3_^−^-N concentration of each treatment had no significant change in SF1 and showed a trend of first increasing and then decreasing (Figure 4b). The peaks in the N160 and N240 treatments were reached on the fourth day, whereas that of the N200 treatment was reached on the fifth day. The NO_3_^−^-N concentrations were always higher in the N200 and N240 treatments than the N160 treatment. The NO_3_^−^-N concentration was higher in the N200 treatment than in the other treatments after the fourth day. The NO_3_^−^-N concentration in the overlying water increased sharply on the seventh day of fertilization in SF2 (Figure 4c). In the first six days, no significant change was found in the N160 treatment and a weak peak appeared on the fourth day, whereas the NO_3_^−^-N concentration in the N200 and N240 treatments increased first and then decreased, and the peak appeared on the second day of fertilization. At the SF2 observation, the average NO_3_^−^-N concentrations were 53% and 87% higher in the N200 and N240 treatments, respectively, than in the N160 treatments.

### 2.5. N_2_O Emissions

**Figure 5:**
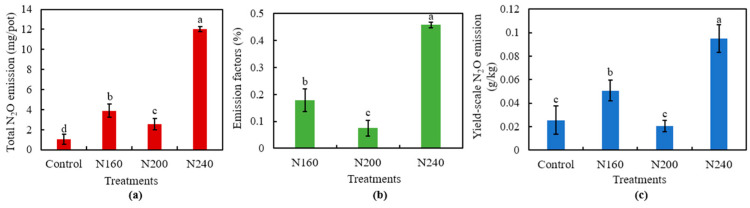
Total N_2_O emissions (**a**), emission factor (**b**), and the yield-scaled N_2_O emissions (**c**) from saline soil with different N application rates. Bars refer to the SD of the mean value (*n* = 3). Different letters above the same column indicate statistically significant differences between treatments, according to Duncan’s post hoc test at the level of *p* < 0.05.

Total N_2_O emissions, emission factors, and the yield-scaled N_2_O emissions all decreased first and then increased with increasing N application (Figure 5). We found that N200 treatment had the lowest total N_2_O emissions, emission factor, and yield-scaled N_2_O emissions. The total N_2_O emissions were higher under all N applications than under the control, but total N_2_O emissions were 34.7% and 78.9% significantly (*p* < 0.05) lower in the N200 treatment than the N160 and N240 treatments, respectively (Figure 5a). Meanwhile, N2O emissions were significantly (*p* < 0.05) lower in the N200 treatment by 57.8% and 83.5%compared to the N160 and N240 treatments, respectively (Figure 5b). The yield-scaled N2O emissions of N200 was similar to that of the control, but was 59.5% and 78.3% significantly (*p* < 0.05) lower than that of N160 and N240, respectively (Figure 5c).

### 2.6. Soil pH, NH_4_^+^-N, and NO_3_^−^-N Content and N Cycling-Related Functional Gene Copies

**Figure 6:**
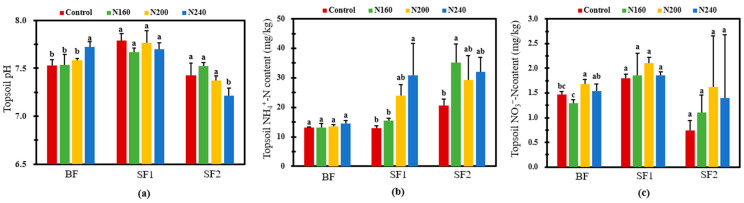
Soil pH (**a**), NH_4_^+^-N (**b**), and NO_3_^—^N (**c**) concentrations after the three stages of fertilization for one week in the rice-growth season with different N application rates. Bars refer to the SD of the mean value (*n* = 3). Different letters above same column indicate statistically significant differences between treatments, according to Duncan’s post hoc test at the level of *p* < 0.05.

Cultivated layer soils had different pHs at the BF, SF1, and SF2 observations (Figure 6a). Overall, there was no difference in soil pH among the treatments with different N inputs. Only the soil in N240 had significantly (*p* < 0.05) higher and lower pHs than those in the other treatments at BF and SF2, respectively. After BF, no change was observed regarding the surface soil NH_4_^+^-N content (Figure 6b). In SF1, the NH_4_^+^-N concentration in the surface soil varied greatly and increased with the increase in N application. In particular, the NH_4_^+^-N content in the surface soil of N240 was 2.4-fold that in the control. The NH_4_^+^-N concentration in the cultivated layer soil decreased first and then increased with the increase in the N application rate in SF2. Although the difference in the surface soil NH_4_^+^-N concentration among the N-added treatments was not significant, the NH_4_^+^-N content of the N160 soil was 70.1% significantly (*p* < 0.05) higher than that of the control. In comparison to the control, no change in surface soil NO_3_^−^-N was found in the three N-added treatments in either the SF1 or the SF2 case (Figure 6c). However, at BF, the surface soil from N200 had significantly (*p* < 0.05) 14.2% higher NO_3_^−^-N than that from N160. In addition, we found that NO_3_^−^-N concentration at each fertilizer stage was the highest in the N200 treatment, and the average NO_3_^−^-N concentration was 26.7% and 12.5% higher in the N200 treatment than in the N160 and N240 treatments, respectively, during the whole growth process of rice.

The AOA *amoA* gene copies were decreased due to the N application (Table 4). In addition, two high N inputs (N200 and N240) resulted in 51.1–62.5% (*p* < 0.05) lower AOA *amoA* gene copies than the low N input (N160). Compared with the control, AOB *amoA* gene copies were increased significantly (*p* < 0.05) in N160, which was 9.0- and 2.3-fold that in N200 and N240, respectively. The N applications at 160 and 200 kg/ha significantly (*p* < 0.05) reduced the copies of the *nirK* gene by 30.4% and 50.7%, respectively, compared with the control. When the N application rate further increased to 240 kg/ha, no change was found when compared to the control. Low N inputs with 160–200 kg/ha exerted no influence on the *nirS* and *nosZ* gene copies. However, they increased dramatically when 240 kg/ha N was applied to the saline soil. That is, the *nirS* and *nosZ* gene copies under N240 were 3.3–4.8- and 3.9–5.2-fold those under the other three experimental treatments.

## 3. Discussion

### 3.1. Effect of Different N Inputs on Rice Yield and N Uptake

Nitrogen is an important limiting factor for crop yield. Different N inputs have different effects on rice yield and yield components [15,23]. Guo et al. (2021) conducted an experiment on the sodic saline–alkaline soils in the western Songnen plain and found that there was no parabolic relationship between rice yield and N application, which reached the peak when the N application rate was 150 kg/ha [24]. The results of the present experiment show that rice yield continued to increase with the increase in the N application rate, which could be attributed to the saline soil used in this experiment having very poor soil fertility. The soil they used only had 9 g/kg organic matter content, far less than the organic matter content (36.2 and 34.6 g/kg) in Guo’s experiment and even lower than the average topsoil organic matter content of Chinese farmland soil (24.82 g/kg) [24,25]. Thus, a higher N application rate is needed to meet the demands of crop growth [26]. Nevertheless, there was no significant difference in rice grain yield between N200 and N240 in the current experiment, and when the N application rate reached 200 kg/ha, a continued increasing N application rate had no significant yield-increase effect. Sui et al. (2013) pointed out that the increased number of effective spikes, kernels per spike, and grain weight contributed the improved rice grain yield [27]. According to the data of rice yield components in the current study, increasing N fertilizer could increase the spike number and kernels per spike and therefore improve the yield. In particular, the kernels per spike reached the highest value of 137.4 under the N200 treatment. The rice SPAD was significantly (*p* < 0.05) higher in the N200 and N240 treatments than in the N160 treatment at the earing stage and the maturation stage (Table 1). The SPAD of rice leaves increased with the increasing N application rate and higher chlorophyll content was an important factor for high rice yield, which is consistent with the results of a split area test on saline–alkali meadow soil by Meng et al. (2022) [28,29,30]. This could be one of the reasons why the increased rice grain yield under saline soils received 200 and 240 kg/ha N. However, the plant height, SPAD, and NDVI of rice in N160 were lower, and the SPAD and NDVI in particular were even lower than the control at the earing stage. Therefore, excessive N reduction would threaten rice growth and lead to a yield decline.

The N uptake by rice was related to the N application rates, and N use efficiency generally decreased with an increase in the N application rate in sodic saline–alkaline rice paddies [24,31]. Our results show that with the increase in the N application rates, N uptake by rice shoot biomass increased and the N use efficiency of rice straw increased. However, the N use efficiency of grain increased first and then decreased, reaching the peak in the N200 treatment. The field experiment on sodic saline–alkali soil at Songnen plain by Lv et al. (2021) showed that the growth rate of stems was higher under low N treatment than that under high N treatment, which is significant for improving the dry matter of stems and prevent lodging at a low N level [31]. In other words, lodging maybe cause the loss of biomass at a high N level. This helps to explain the higher rice N use efficiency of N200 in comparison with N240. In addition, excessive N application increased nutrient reserves in vegetative organs [32] and increased the N proportion in straw but in turn reduced the grain N use efficiency. According to data in Table 1 and Table 2, the rice grain yield was similar between the N240 and N200 treatments, but the straw biomass was significantly (*p* < 0.05) higher in the N240 treatment compared to the N200 treatment, and the rice harvest index decreased, leading to a decrease in grain N use efficiency.

Soil NO_3_^−^-N content is an important determinant of the N application rate and increased with the increase in the N application rate [33,34]. After N reduction, soil NO_3_^−^-N content and N loss decreases and N use efficiency increases. In this experiment, the NO_3_^−^-N content in each fertilizer stage increased first and then decreased with the increase in the N application rate and reached the maximum at 200 kg/ha, which is inconsistent with the results of most previous studies that were conducted in non-saline soils [33,34]. The reason could be that the relatively low N application rate in saline soil is not enough to support the normal growth of plants, which leads to a decrease in NO_3_^−^-N reductase activity in plants and an increase in NO_3_^−^-N accumulation [35]. Therefore, the residual NO_3_^−^-N in soil also increases after crop harvest, which may lead to higher soil NO_3_^−^-N content in N200 than in N240. In addition, based on the results from a pot simulation trial under different soil salinity, Min et al. (2012) reached a similar conclusion that soil NO_3_^−^-N content was the highest at the medium N level, followed by a high N level [36]. However, there was no significant difference in NO_3_^−^-N content between the N240 and N200 treatments in this experiment. Therefore, comprehensively considering the grain yield, N uptake, and use efficiency of the crop, the optimal recommended N application rate is 200 kg/ha in saline soil according to the current work.

### 3.2. Effect of N Application at Varied Rates on NH_3_ Volatilization from Saline Soil

Soil NH_3_ volatilization in paddy fields increased linearly with the increase in the N application rate. Furthermore, in saline soil with elevating salinity, suppressed urea hydrolysis, nitrification, and soil NH_4_^+^-N adsorption capacity could promote NH_3_ volatilization [37,38]. The present results show that the total NH_3_ volatilization increased with the increase in the N application rate during the whole rice-growth cycle. Similar NH_3_ volatilization patterns were observed in rice cultivation under slightly and moderately salt-affected soil [39]. However, in actual production, excessive N reduction in order to reduce NH_3_ volatilization may result in decreased crop production. Therefore, NH_3_ volatilization and crop yield should be considered of equal importance, so the yield-scaled NH_3_ volatilization is of more practical significance [40]. However, unlike in non-saline agricultural soil, there are few studies on the yield-scaled NH_3_ volatilization of rice production in saline–alkali soil [41]. In this study, the yield-scaled NH_3_ volatilization decreased first and then increased with the increase in the N application rate, and the lowest value was found in the N200 treatment.

In terms of the fertilization stage, NH_3_ volatilization during SF1 accounted for a higher proportion under the different N application rates than that during BF and SF2 in this experiment. The temperature recorded at SF1 was relatively higher and the activity of urease was enhanced, which promoted urea hydrolysis and intensified NH_3_ volatilization [42]. Therefore, reducing NH_3_ volatilization in SF1 is key to reducing the total NH_3_ volatilization. In our experiment, the NH_3_ volatilization ratio in SF1 was at the maximum in the N200 and N240 treatments. This is consistent with the laboratory simulation test results of Li (2017), which indicated that the maximum NH_3_ volatilization ratio of rice in saline–alkali soil was in SF1 with an N application rate of 75–300 kg/ha [43]. As for other two stages, the NH_3_ volatilization load during BF under the N240 treatment was higher than during SF2, which is similar to the experiment conducted by Wang et al. (2012) in non-saline soil [44]. This may be due to the sparse growth of rice in BF and a root system that was insufficient for absorbing N, resulting in a greater loss of NH_3_ volatilization, whereas the growth of rice in SF2 was vigorous, and the rice plants directly blocked gas flow and reduced NH_3_ volatilization [45,46].

The pH and NH_4_^+^-N in the overlying water of paddies are important indicators to regulate N migration and transformation in saline–alkali paddy ecosystems [47]. From the perspective of the whole rice-growth cycle, the dynamic pattern of pH and NH_4_^+^-N in the overlying water was similar, which increased first and then decreased. The NH_3_ volatilization boosted with the increase in pH in the overlying water, an effect that was more significant for the BF observation [48]. For the three N application rates, the N200 treatment recorded the highest pH values in the BF and SF1 phases, but the highest NH_3_ volatilization was found in the N240 treatment. This may be due to NH_4_^+^ itself tending to transform in the direction of NH_3_ in saline soil with a high pH, resulting in a different variation trend of pH and NH_3_ volatilization in overlying water [49]. Reducing the concentration of NH_4_^+^-N in the overlying water is a key approach to reducing the NH_3_ volatilization [50]. Wang et al. (2023) found that the NH_4_^+^-N concentration in the overlying water was significantly positively correlated with NH_3_ volatilization in saline–alkali paddy fields [47]. In the current experiment, NH_3_ volatilization in SF1 accounted for a relatively large proportion, and the concentration of NH_4_^+^-N in the overlying water in SF1 was higher in N240 than in other N-applied treatments, which confirms the results of previous studies.

### 3.3. Effect of Different N Applications on N_2_O Emissions

Nitrification and denitrification processes with the participation of soil microorganisms produced the N_2_O. The application of N fertilizer can significantly increase NH_4_^+^-N and NO_3_^−^-N concentration in soil, which could enhance the nitrification and denitrification capacities and consequently promote soil N_2_O emissions [51,52,53]. In saline soils, the imbalance of salinity inhibition on nitrite (NO_2_^−^-N) oxidation and NH_3_ oxidation leads to the accumulation of NO_2_^−^-N in soil, which leads to an increase in N_2_O emissions. In addition, compared with non-salt soil, the decrease in microbial respiration in saline–alkali soil also reduces the oxygen consumption in the overlying water and/or surface layer soil and increases the dissolved oxygen, which inhibits the synthesis of N_2_O reductase and reduces the abundance of the *nosZ* gene, further leading to an increase in N_2_O emissions [38,54,55,56]. A previous study has shown that application of urea significantly promoted cumulative N_2_O emissions in saline–alkali soil [47]. In this experiment, the N_2_O emissions did not continuously increase with the increase in the N application rate and reached the minimum when the N application rate was 200 kg/ha. The change in soil pH affects denitrification enzyme activity, which then affects N_2_O emissions. With the increase in the soil pH, the activity of soil N_2_O reductase also increases [57]. The soil pH of the N240 treatment was lower than that of the N200 treatment in SF1 and SF2 in this experiment, which led to low N_2_O reductase activity and may be a reason why N_2_O emissions in the N240 treatment were higher than those in the N200 treatment. Similarly, Jia et al. (2020) reported that N_2_O emissions were nonlinearly related to the N application rate under salt stress [58].

Nitrogen application affected the soil pH and the abundance of the N cycle functional gene, such as the nitrification process of functional genes AOA and AOB *amoA* and the denitrification process of functional genes *nirK*, *nirS*, and *nosZ* [59]. Our results show that the gene copies of AOA *amoA*, AOB *amoA*, *nirS*, and *nirK* in soil decreased first and then increased with the increase in the N application rate one week after SF2 (Table 4), which is similar to the changes in N_2_O emissions, and all reached the lowest point in the N200 treatment. However, the *nosZ* gene copies increased with the increase in the N application rate, which is different from the change trend of N_2_O emissions. Ammonia oxidation to NO_2_^−^-N is the ring-limiting step of nitrification, so the control of microorganisms (AOA and AOB) in this process is an important measure to control N_2_O emissions [60]. In the current study, the AOA and AOB *amoA* gene copies under the N200 treatment were the lowest, resulting in the lowest N_2_O emissions. Shi et al. (2019) also proved that AOB is the main contributor to N_2_O emissions through experiments on saline–alkali soil [61]. The symbolic process of denitrification is the reduction of NO_2_^−^-N to NO, which is mainly completed by the nitrite reductase *nirK* and *nirS* genes [62], which may be the reason why N_2_O emissions are correlated with an abundance of *nirK* and *nirS* genes. Pan et al. (2023) pointed out that the increase in urea significantly increased the denitrification rate and slowed down the decrease in *nirK* gene abundance due to saline–alkali treatment but had no significant effect on *nirS* gene abundance [63], which is similar to the results of this study. The *nirK* gene abundance in the N240 treatment was higher than that in the N200 treatment, whereas there was no difference in the *nirS* gene abundance between the N160 and N200 treatments. The increased ratio of (*nirK* + *nirS*)/*nosZ* in the saline soil environment indicates that the ratio of the final N_2_O/(N_2_O + N_2_) product increased, and thus the genetic potential of N_2_O emission was improved [64]. The (*nirK* + *nirS*)/*nosZ* ratio in the N200 treatment was the lowest in this experiment, which may have led to it having the lowest N_2_O emissions.

## 4. Materials and Methods

### 4.1. Experiment Setup

#### 4.1.1. Soil Properties

The paddy soil column simulation test was carried out in a greenhouse of Jiangsu Academy of Agricultural Sciences (32°08′ N, 118°82′ E) in an East Asian monsoon climate zone with an annual precipitation of 1106.5 mm and an average annual temperature of 15.5 °C. The tested soil was clay and had the following basic physicochemical properties: an initial pH (1:2.5 soil to water) of 8.01, a total salt content of 4.4‰, a total N content of 0.69 g/kg, an available P content of 4.72 mg/kg, an available K content of 147.47 mg/kg, a soil organic matter content of 9.00 g/kg, a cation-exchange capacity of 2.54 mg/kg, and 21.67% sand, 20.67% silt, and 56.67% clay.

#### 4.1.2. Experimental Design and Management

In this experiment, four treatments were set up according to different N application rates: 0, 160, 200, and 240 kg/ha, denoted as control, N160, N200, and N240, respectively. No N was applied as the control and each treatment was replicated three times. N, phosphorus (P), and potassium (K) fertilizers were arranged using urea (46% N), calcium superphosphate (12% P_2_O_5_), and potassium chloride (60% K_2_O), respectively. The input of P and K fertilizers (in P_2_O_5_ and K_2_O, respectively) was 90 and 120 kg/ha for each treatment, respectively. N fertilizers were applied as 30%, 30%, and 40% of the basal N fertilization stage (BF), first supplement N fertilization stage (SF1), and second supplement N fertilization stage (SF2), respectively, whereas P and K fertilizers were applied in one application of BF. The soil columns were made of PVC material with a height of 50 cm and a diameter of 30 cm, and the amount of N, P, and K fertilizer applied in the soil columns was converted to 0.07 m^2^ per column. Rice (*Oryza sativa* L., var. Nangeng 46) was transplanted in June 2022, with three holes per soil column and three seedlings per hole, and was harvested in November.

### 4.2. Sample Collection and Determination

#### 4.2.1. Rice Growth, Yield, and N Use Efficiency

During the rice-growth season, the Normalized Difference Vegetative Index (NDVI) values were measured with a hand-held GreenSeeker (Trimble, Westminster, CO, USA) crop sensor. The Soil Plant Development Analysis (SPAD) values were measured with a SPAD 502 (Konica Minolta, Tokyo, Japan) meter. The straw and grain were harvested separately at the maturation stage, the spike number and kernels per spike in each soil column were recorded, and the weight of the straw and grain was measured. The dried straw and grain samples were digested with H_2_SO_4_ and H_2_O_2_ and the total N content was measured by the Kjeldahl method [65]. Harvest index, straw and grain N uptake, and N use efficiency were calculated according to the following formulas.

(1) $$\mathrm{Harvest}\;\mathrm{index}\;\left(\%\right)=\frac{\mathrm{dried}\;\mathrm{grain}\;\mathrm{weight}\;\left(g\right)}{\mathrm{dried}\;\mathrm{grain}\;\mathrm{weight}\;(g)+\mathrm{dried}\;\mathrm{straw}\;\mathrm{weight}\;(g)}\times 100\%\;$$

(2) $$\mathrm{Straw}\;\left(\mathrm{grain}\right)\;N\;\mathrm{uptake}\left(g\right)=\mathrm{dry}\;\mathrm{weight}\;\mathrm{of}\;\mathrm{straw}\;\left(\mathrm{grain}\right)\;(g)\;\times \;N\;\mathrm{content}\;\mathrm{of}\;\mathrm{straw}\;\left(\mathrm{grain}\right)\left(g/\mathrm{kg}\right)/1000$$

(3) $$N\;\mathrm{use}\;\mathrm{efficiency}\;\left(\%\right)=\frac{N\;\mathrm{uptake}\;\mathrm{in}\;N\;\mathrm{application}\;\mathrm{treatment}\;(g)\;-\;N\;\mathrm{uptake}\;\mathrm{in}\;\mathrm{control}\;(g)}{N\;\mathrm{application}\;\mathrm{rate}\;(g)}\times 100\%$$

#### 4.2.2. NH_3_ Volatilization

NH_3_ volatilization was measured with a modified continuous airflow enclosure method [66]. In this experiment, the chamber was 15 cm in internal diameter and 20 cm in height; the NH_3_ absorbent was 2% boric acid; 20 mL of a mixture of methyl red, bromocresol green, and ethanol were added to each 1 L of absorbent; and 80 mL of NH_3_ absorbent was poured into the absorbent bottle each time. After collection, the chamber was removed from the airtight room to avoid environmental influences and the NH_3_ absorbent solution was immediately titrated by 0.02 mol/L H_2_SO_4_ to calculate the daily NH_3_ volatilization, with the total NH_3_ volatilization being the sum of the daily NH_3_ volatilization during the observation period.

(4) $$\mathrm{The}\;\mathrm{yield}-\mathrm{scaled}\;{\mathrm{NH}}_{3}\;\mathrm{volatilization}\;(g/\mathrm{kg})=\frac{\mathrm{total}\;{\mathrm{NH}}_{3}\;\mathrm{volatilization}\;(g)}{\mathrm{grain}\;\mathrm{yield}\;(\mathrm{kg})}$$

(5) $$\mathrm{Emission}\;\mathrm{factor}\;\left(\%\right)=\frac{{\mathrm{NH}}_{3}\;\mathrm{volatilization}\;\mathrm{in}\;N\;\mathrm{treatment}\;(g)\;-\;{\mathrm{NH}}_{3}\;\mathrm{volatilization}\;\mathrm{in}\;\mathrm{control}\;(g)}{N\;\mathrm{application}\;\mathrm{rate}\;(g)}\times 100\%$$

#### 4.2.3. N_2_O Emissions

N_2_O was determined by the modified closed chamber method [66]. Four consecutive gas samples were collected on the second, fourth, sixth, and eighth days after fertilization and every 10–12 days thereafter, but gas samples were collected on the second, fourth, sixth, and eighth days after the start of the mid-season drainage period. When sampling, a transparent Plexiglas cylinder (inner diameter 36 cm, height 100 cm) was placed in the water tank to form a closed space. The gas in the chamber was collected for 15, 30, 45, and 60 min, and the temperature in the chamber was recorded. Gas samples were analyzed and determined with a gas chromatograph (Agilent 7890A, Agilent Technologies, Santa Clara, CA, USA). The N_2_O emissions were calculated according to the following formula, and the total N_2_O emissions was the accumulation of N_2_O emissions collected every time throughout the whole rice season.

(6) F=ρ × h ×∆c/∆t×273/T

Note: F indicates the N_2_O emissions (µg/m^2^/h), ρ indicates the N_2_O gas density in standard conditions (1.25 kg/m^3^), h indicates the transparent Plexiglas cylinder height, ∆c/∆t indicates the rate of change of gas concentration in the transparent Plexiglas cylinder with time (µg/L/h), and T indicates the absolute temperature inside the transparent Plexiglas cylinder.

(7) $$\mathrm{Emission}\;\mathrm{factor}\;\left(\%\right)=\frac{N_{2}O\;\mathrm{emission}\;\mathrm{in}\;N\;\mathrm{treatment}\;(g)\;-\;N_{2}O\;\mathrm{emission}\;\mathrm{in}\;\mathrm{control}\;(g)}{N\;\mathrm{application}\;\mathrm{rate}\;(g)}\times 100\%$$

(8) $$\mathrm{The}\;\mathrm{yield}-\mathrm{scaled}\;N_{2}O\;\mathrm{emission}\;(g/\mathrm{kg})=\frac{\mathrm{total}\;N_{2}O\;\mathrm{emission}(g)}{\mathrm{rice}\;\mathrm{grain}\;\mathrm{yield}\;(\mathrm{kg})}$$

#### 4.2.4. The pH, NH_4_^+^-N, and NO_3_^−^-N Concentrations of Overlying Water

We collected approximately 100 mL of overlying water on days 1, 3, 5, and 7 after fertilizer application and immediately brought them back to the laboratory for freezing. The pH was determined in situ using a convenient pH meter. The concentrations of NH_4_^+^-N and NO_3_^−^-N in the overlying water were determined by indophenol blue colorimetry and an ultraviolet spectrophotometer.

#### 4.2.5. The pH, NH_4_^+^-N, and NO_3_^−^-N Concentrations and Copies of N Cycle Functional Genes in Soils

Soil was collected from the 0–20 cm soil layer during the basal N fertilization stage, first supplement N fertilization stage, and second supplement N fertilization stage; air dried; and sieved; and the fresh soil samples were frozen and stored in the laboratory refrigerator for use. A total of 10 g of air-dried soil sample was taken, 25 mL of water were added to stand for half an hour, and then a pH meter was used to determine the soil pH. A total of 5 g fresh soil samples was extracted by adding 25 mL of 2 mol/L KCl solution and then filtrated after shaking for 1 h. NH_4_^+^-N and NO_3_^−^-N were determined by indophenol blue colorimetry and an ultraviolet spectrophotometer [67]. The abundance of soil N cycle functional genes (AOB *amoA*, AOA *amoA*, *nirS*, *nirK*, and *nosZ*) was quantitatively determined by qPCR [68].

### 4.3. Statistical Analysis

The data were collated and analyzed by ANOVA using Excel 2010 and SPSS 16.0 software (SPSS Inc., Chicago, IL, USA). Multiple comparison tests were performed between the different treatments using Duncan’s method, with different lowercase letters indicating significant differences between treatments at the significance level of *p* < 0.05.

## 5. Conclusions

The rice grain yield increased with the increase in the N application rate in a certain range, but when the N application rate was higher than 200 kg/ha, the grain yield was not further improved in the saline soil. The kernels per spike reached the maximum in N200. More N input induced higher total NH_3_ volatilization from the rice-growth cycle. The NH_4_^+^-N concentration of the overlying water in SF1 and SF2 were both at a low level in the N200 treatment. Therefore, we obtained the lowest yield-scaled NH_3_ volatilization (3.3 g/kg) at a medium N input (200 kg/ha). The gene copies of AOA *amoA*, AOB *amoA*, *nirS*, and *nirK* in soil decreased first and then increased with the increase in the N application rate. Meanwhile, both total N_2_O emissions and the yield-scaled N_2_O emissions reached the minimum in the N200 treatment. Considering the responses of rice yield, NH_3_ volatilization, and N_2_O emissions to different N application rates, 200 kg/ha is recommended to achieve a higher crop yield and lower environmental N losses in reclaimed saline soils. Meanwhile, the long-term effects of different N managements on crop production and N losses from saline soil should be clarified at the field scale in future investigations.

## Figures and Tables

**Table 1:** Rice straw biomass, grain yield, and the related agronomic traits of rice grown in saline soil received with different N inputs.

Treatment	Straw Biomass (g/pot)	Grain Yield (g/pot)	Spike Number	Kernels per Spike	Thousand-Kernel Weight (g)	Harvest Index (%)
Control	82.7 ± 7.9 d	40.1 ± 1.5 c	15.0 ± 1.0 d	100.8 ± 0.9 c	24.9 ± 0.5 ab	32.8 ± 1.3 b
N160	125.7 ± 9.8 c	76.7 ± 4.1 b	28.7 ± 1.5 c	117.6 ± 8.0 b	24.1 ± 0.1 b	37.9 ± 2.6 a
N200	207.9 ± 12.7 b	123.7 ± 1.8 a	39.3 ± 3.5 b	137.4 ± 12.2 a	25.5 ± 0.5 ab	37.3 ± 1.1 a
N240	269.6 ± 28.5 a	127.8 ± 13.5 a	45.5 ± 1.5 a	125.2 ± 6.8 ab	25.6 ± 1.3 a	32.2 ± 0.7 b

Note: Data are presented as mean ± SD (*n* = 3). Different letters in same column indicate statistically significant differences between treatments, according to Duncan’s post hoc test at the level of *p* < 0.05.

**Table 2:** N content and uptake of rice straw and grain grown in saline soils with different N application rates.

Treatment	N Content (g/kg)		N Uptake (g/pot)		
	Rice Straw	Rice Grain	Rice Straw	Rice Grain	Total
Control	4.39 ± 0.01 b	18.20 ± 0.10 c	0.36 ± 0.03 c	0.73 ± 0.03 c	1.09 ± 0.06 d
N160	4.82 ± 0.18 b	19.40 ± 0.41 bc	0.61 ± 0.05 c	1.49 ± 0.07 b	2.09 ± 0.07 c
N200	6.74 ± 0.93 a	21.53 ± 1.10 a	1.41 ± 0.28 b	2.66 ± 0.11 a	4.07 ± 0.25 b
N240	7.14 ± 0.19 a	20.31 ± 1.09 ab	2.03 ± 0.27 a	2.70 ± 0.28 a	4.74 ± 0.54 a

Note: Data are presented as mean ± SD (*n* = 3). Different letters in same column indicate statistically significant differences between treatments, according to Duncan’s post hoc test at the level of *p* < 0.05.

**Table 3:** NH_3_ volatilization, emission factors, and yield-scaled NH_3_ volatilization in the rice-growth cycle from saline soil with varied N inputs.

Treatment	NH_3_ Volatilization (g/pot)				Emission Factor (%)	Yield-Scaled NH_3_ Volatilization (g/kg)
	BF	SF1	SF2	Total		
Control	0.02 ± 0.00 c	0.01 ± 0.00 b	0.03 ± 0.01 c	0.06 ± 0.01 c	–	1.31 ± 0.09 c
N160	0.07 ± 0.01 b	0.13 ± 0.00 ab	0.15 ± 0.02 a	0.35 ± 0.02 b	26.01 ± 2.08 a	4.57 ± 0.30 a
N200	0.09 ± 0.01 b	0.22 ± 0.11 a	0.10 ± 0.01 b	0.41 ± 0.11 b	24.98 ± 7.55 a	3.30 ± 0.81 b
N240	0.20 ± 0.02 a	0.27 ± 0.11 a	0.13 ± 0.02 ab	0.59 ± 0.14 a	31.63 ± 8.52 a	4.47 ± 1.09 a

Note: Data are presented as mean ± SD (*n* = 3). Different letters in same column indicate statistically significant differences between treatments, according to Duncan’s post hoc test at the level of *p* < 0.05. BF, SF1, and SF2 refer to the basal, first, and second supplementary N fertilizations, respectively.

**Table 4:** The soil N cycling-related functional gene copies in saline soil collected at one week after SF2 applied in the rice-growth season with different N inputs.

Treatment	AOA *amoA*	AOB *amoA*	*nirK*	*nirS*	*nosZ*
	10^4^ Copies/g		10^6^ Copies/g	10^7^ Copies/g	10^7^ Copies/g
Control	24.17 ± 0.62 a	24.37 ± 4.49 bc	8.73 ± 0.49 a	3.02 ± 0.09 b	0.59 ± 0.01 b
N160	16.82 ± 4.36 b	102.24 ± 26.48 a	6.08 ± 0.10 b	4.00 ± 0.56 b	0.57 ± 0.08 b
N200	6.30 ± 1.27 c	11.34 ± 3.82 c	4.30 ± 0.23 c	2.72 ± 0.53 b	0.77 ± 0.19 b
N240	8.85 ± 1.35 c	44.50 ± 1.58 b	8.61 ± 1.03 a	13.12 ± 2.12 a	2.98 ± 0.35 a

Note: Data in the table are presented as mean ± SD (*n* = 3). Different letters in same column indicate statistically significant differences between treatments, according to Duncan’s post hoc test at the level of *p* < 0.05.

## Data Availability

Data are available after requesting.
